# The Prevalence of Sleep Disorders in Populations with Glymphatic Dysfunction: A Systematic Review and Meta-Analysis

**DOI:** 10.3390/biology15040309

**Published:** 2026-02-10

**Authors:** Zaw Myo Hein, Che Mohd Nasril Che Mohd Nassir, Hafizah Abdul Hamid, Muhammad Farris Iman Leong Abdullah, Nisha Shantakumari

**Affiliations:** 1 Department of Basic Medical Sciences, College of Medicine, Ajman University, Ajman P.O. Box 346, United Arab Emirates; z.hein@ajman.ac.ae; 2 Center of Medical and Bio-Allied Health Sciences Research (CMBHSR), Ajman University, Ajman P.O. Box 346, United Arab Emirates; 3 Department of Anatomy and Physiology, Faculty of Medicine, Universiti Sultan Zainal Abidin, Kuala Terengganu 20400, Terengganu, Malaysia; 4 Department of Human Anatomy, Faculty of Medicine and Health Sciences, Universiti Putra Malaysia, Serdang 43400, Selangor, Malaysia; a_hafizah@upm.edu.my; 5 Department of Psychiatry and Mental Health, Faculty of Medicine, Universiti Sultan Zainal Abidin, Kuala Terengganu 20400, Terengganu, Malaysia; farrisiman@unisza.edu.my

**Keywords:** sleep disorders, glymphatic dysfunction, prevalence, meta-analysis, neurodegeneration, brain clearance, preventive neurology

## Abstract

The glymphatic system supports metabolic waste clearance during sleep and is essential for brain homeostasis. Disruption of this system has been linked to sleep disorders, yet the overall prevalence of sleep disorders in populations showing impaired glymphatic-related function remains unclear. This systematic review and meta-analysis evaluated the prevalence of sleep disorders in human cohorts with structural, functional, or biochemical imaging markers of impaired glymphatic activity. Following PRISMA guidelines, major databases were searched up to August 2025. Eligible observational studies reported sleep disorder prevalence in populations characterized by enlarged perivascular spaces, white matter hyperintensities, DTI-ALPS (DTI-ALPS: Diffusion tensor image analysis along perivascular space) alterations, ultrafast fMRI (fMRI: functional magnetic resonance) indices, or CSF/PET (CSF: cerebrospinal fluid; PET: positron emission tomography) clearance deficits. Random-effects models generated pooled estimates, and heterogeneity, publication bias, and moderators were examined using I^2^ statistics, Egger’s test, trim-and-fill, and meta-regression. Nineteen studies (≈4500 participants) met the inclusion criteria. The pooled prevalence of sleep disorders in populations with impaired glymphatic-related function was 44.9% (95% CI: 34.9–55.3%), with substantial heterogeneity (I^2^ ≈ 95%). Meta-regression identified older age and case–control design as significant contributors, while imaging modality, sex distribution, and sample size were not. Publication bias was minimal. Sleep disorders are common among individuals with impaired glymphatic-related markers, reflecting co-occurrence rather than causality. Standardized longitudinal studies are needed to clarify mechanisms and clinical relevance.

## Simple Summary

Good sleep is essential for keeping the brain healthy. During sleep, the brain uses a natural cleaning system to remove waste products that can damage brain cells if they build up over time. Problems with this cleaning process have been linked to brain diseases, but it is still unclear how common sleep disorders are in people who show signs of poor brain waste clearance. In this study, we reviewed and combined results from many previous human studies to answer this question. We found that sleep disorders are very common in people with signs of impaired brain waste removal, affecting almost one in two individuals. This rate is much higher than is usually seen in the general population. Our findings suggest that sleep problems and poor brain cleaning often occur together and may worsen each other. Understanding this link is important because improving sleep could help protect brain health, reduce the risk of memory problems, and support healthy ageing. These results highlight the value of early sleep screening and sleep care as part of strategies to prevent brain diseases.

## 1. Introduction

Sleep is a fundamental biological process essential for maintaining cognitive, emotional, metabolic, and immune functions. Despite occupying nearly one-third of human life, its restorative role is often underappreciated. Disruptions in sleep quality or quantity, collectively termed sleep disorders, are increasingly prevalent and represent a major global health burden [1,2]. Epidemiological surveys indicate that 33–50% of adults experience at least one symptom of a sleep disorder, such as insomnia, obstructive sleep apnea (OSA), hypersomnia, or circadian rhythm disturbances [3,4,5,6]. These conditions not only impair daytime performance but also elevate the risk of cardiovascular disease, metabolic syndrome, psychiatric disorders, and neurodegenerative conditions.

Over the past decade, growing neuroimaging and experimental evidence have revealed an intimate bidirectional relationship between sleep and the glymphatic system, a glia-dependent perivascular network responsible for clearing metabolic and neurotoxic waste from the brain. The glymphatic pathway facilitates cerebrospinal fluid (CSF)–interstitial fluid (ISF) exchange and enables the removal of amyloid-β (Aβ), tau, and α-synuclein, key proteins implicated in Alzheimer’s disease (AD), Parkinson’s disease (PD), and related neurodegenerative disorders [7,8]. Several imaging and biomarker approaches have been used to infer glymphatic-related functions. Structural proxies such as enlarged perivascular spaces (ePVS) reflect impaired perivascular clearance [9], while functional techniques, such as diffusion tensor imaging along the perivascular space (DTI-ALPS), estimate fluid transport along perivascular pathways, serving as indirect indicators of glymphatic activity [10].

Physiological studies have demonstrated that glymphatic clearance is markedly enhanced during deep slow-wave sleep (SWS), when reduced noradrenergic tone expands the interstitial space and promotes efficient CSF–ISF exchange [11,12]. Conversely, sleep deprivation acutely suppresses glymphatic transport [13], whereas chronic sleep disruption accelerates protein accumulation, neuroinflammation, and cognitive decline [14]. Sleep disorders such as OSA may further impede glymphatic flow through intermittent hypoxia, oxidative stress, and alterations in cerebral perfusion and perivascular pulsatility [15]. Collectively, these findings suggest a close interdependence between sleep physiology and glymphatic homeostasis.

Despite strong mechanistic support from animal and imaging studies, the human evidence linking sleep pathology and glymphatic-related dysfunction remains fragmented. Most available studies are small, condition-specific, or conducted in narrowly defined clinical cohorts. Moreover, diverse modalities, including MRI-based perivascular space quantification [16], DTI-ALPS metrics [17], ultrafast functional MRI [18], and CSF or PET-based clearance indices [19], capture different, partially overlapping aspects of glymphatic function. Integrating these heterogeneous findings is essential to clarify whether impaired glymphatic-related function is consistently associated with a higher prevalence of sleep disorders across populations.

Therefore, this systematic review and meta-analysis aimed to synthesize current evidence on the prevalence of sleep disorders among individuals with evidence of impaired glymphatic-related function, as determined by structural, functional, or biochemical proxies. Specifically, we sought to (i) estimate the pooled prevalence of sleep disorders across diverse populations and glymphatic-related assessment methods, (ii) explore potential moderators contributing to between-study variability, and (iii) contextualize these findings within mechanistic frameworks linking sleep and glymphatic-related functions. By integrating quantitative estimates with conceptual insights, this review provides a comprehensive foundation for future research on early detection, risk stratification, and prevention of neurodegenerative and cerebrovascular diseases associated with glymphatic-related impairment.

## 2. Materials and Methods

### 2.1. PICOS, Inclusion and Exclusion Criteria

The eligibility criteria for the present systematic review and meta-analysis were established according to the Population, Intervention (or Exposure), Comparison, Outcomes, and Study design (PICOS) framework [19]. These criteria were determined a priori to ensure methodological transparency and reproducibility. A summary of the PICOS framework is presented in Table 1.

The inclusion criteria for this review were (i) studies involving human participants of any age from community-based or clinical cohorts across all geographic regions, (ii) studies reporting sleep disorders (and their categories) with a specific focus on glymphatic-related systems and their proxies, such as ePVS, assessed by MRI of any field strength (≥1.0 T) or MRI-based neuroimaging. However, given the absence of a universally accepted in vivo gold standard for measuring glymphatic clearance in humans, we adopted an inclusive framework of “glymphatic-related dysfunction” by pooling studies using functional indices of perivascular fluid transport (DTI-ALPS, ultrafast fMRI, CSF/PET clearance markers) and structural correlates of impaired clearance (ePVS and WMHs). Although these measures are not biologically equivalent, they reflect partially overlapping aspects of brain clearance failure along a continuum from altered fluid dynamics to downstream tissue injury. Pooling across modalities was therefore undertaken as an exploratory synthesis of converging evidence linking sleep disorders to impaired brain clearance-related processes, rather than as a definitive estimate of glymphatic transport per se. Modality-specific subgroup analyses were not feasible due to the limited number of eligible studies per biomarker. (iii) We considered cross-sectional, cohort (baseline prevalence/occurrence data), and population-based studies, as well as large case series with at least 30 participants, if prevalence/occurrence could be extracted. To qualify, studies had to report occurrence/prevalence estimates of sleep disorders, particularly in relation to glymphatic system dysfunction, or provide sufficient data (including both the numerator and denominator) to calculate occurrence/prevalence. Only peer-reviewed journal articles published in English or other languages with English abstracts and extractable data were considered, with publication year from the inception to 13 August 2025.

The exclusion criteria comprised (i) animal or in vitro studies, (ii) pediatric-only case reports without imaging-based glymphatic system-defined prevalence, and (iii) studies lacking sufficient data to calculate prevalence rates, studies reporting only subjective sleep complaints without standardized diagnostic criteria or validated measurement tools; and studies without any glymphatic-related assessment when aiming to examine the association between sleep disorders and glymphatic dysfunction. In terms of study design, case reports, case series with fewer than 30 participants, interventional trials without baseline prevalence data, reviews, editorials, letters, and conference abstracts without extractable data were not considered. We further excluded studies that did not report prevalence or provide sufficient data to calculate it, as well as non-peer-reviewed sources (e.g., theses, proceedings) and non-English publications without extractable occurrence/prevalence data.

### 2.2. Search Strategy and Study Selection

**Figure 1:**
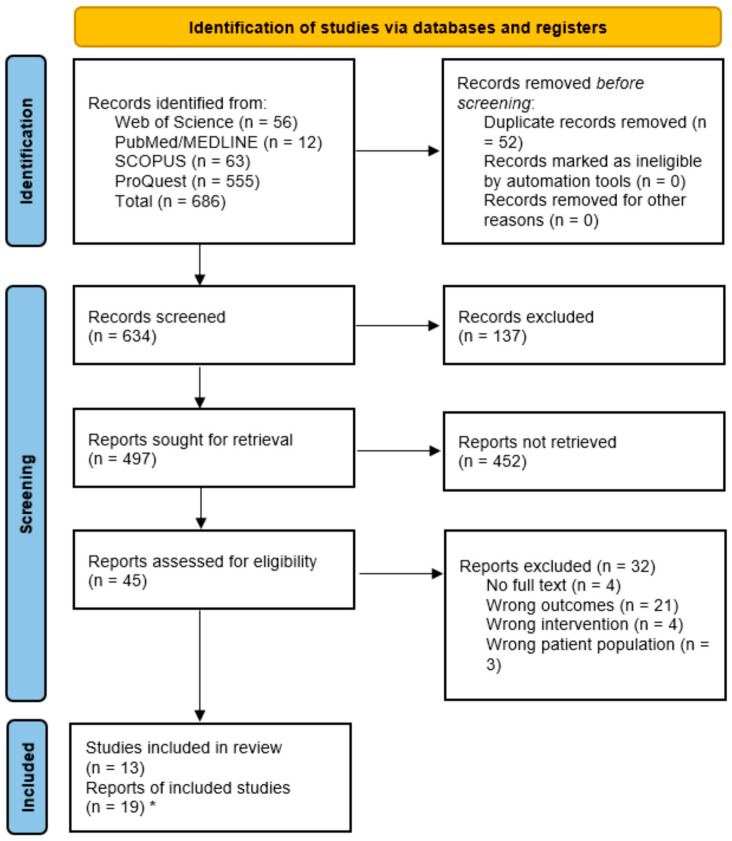
Search strategy flow diagram based on PRISMA 2020 guideline for new systematic reviews, which included searches of databases, registers, and other sources. * Six additional eligible studies were identified during data extraction and quantitative analysis (through reference list screening and cross-checking) and were included in the final meta-analysis.

A comprehensive literature search was conducted using four major online databases: (i) PubMed (National Center for Biotechnology Information), (ii) Web of Science core collection, which includes records from the Science Citation Index Expanded (SCI-EXPANDED) and the Emerging Sources Citation Index (ESCI), (iii) Scopus, and (iv) ProQuest. The search protocol was developed following the 2020 Preferred Reporting Items for Systematic Reviews and Meta-Analyses (PRISMA) guidelines, and the process is illustrated in the PRISMA flow diagram (Figure 1). Methodological decisions were informed by previously published systematic reviews and meta-analyses in related fields [20,21].

The search strategy was designed to identify studies reporting the prevalence of sleep disorders, with a specific focus on those assessing glymphatic functions directly or indirectly. Both controlled vocabulary (e.g., Medical Subject Headings [MeSH] in PubMed) and free-text terms were employed. Boolean operators “AND” and “OR” were used to combine search terms, which were grouped into three main keyword categories: (i) sleep disorder categories: insomnia, sleep-related breathing disorders, central disorders of hypersomnolence, circadian rhythm sleep–wake disorders, parasomnias, and sleep-related movement disorders. Keywords 1 (sleep disorders): “sleep disorders” OR insomnia OR “sleep-related breathing disorders” OR “central disorders of hypersomnolence” OR “circadian rhythm sleep-wake disorders” OR parasomnias OR “sleep-related movement disorders”. Keywords 2 (epidemiological measures): prevalence OR epidemiology OR frequency OR burden. Keywords 3 (glymphatic function): glymphatic OR “glymphatic system” OR “Virchow-Robin space” OR “perivascular spaces” OR PVS OR ePVS OR “meningeal lymphatic”.

The combined search string was structured as: (Keywords 1) AND (Keywords 2) AND (Keywords 3), as outlined in Supplementary Table S1. No publication year and language restrictions were applied, and only human studies were considered. All retrieved records were imported into EndNote X9 (Clarivate Analytics, Philadelphia, PA, USA) for reference management. Duplicates were removed using EndNote and were confirmed by Zotero Reference Manager (Version 6.0; Corporation for Digital Scholarship, Vienna, VA, USA) and Covidence systematic review software (Veritas Health Innovation, Melbourne, Australia). Screening was performed in two stages: (i) Title and abstract screening, conducted independently by two reviewers to exclude irrelevant records, and (ii) Full-text screening, to determine final eligibility according to the inclusion and exclusion criteria. Discrepancies at either stage were resolved by consensus or, when necessary, by consultation with a third reviewer. During title–abstract screening, Reviewer A (CMNCMN) and Reviewer B (ZMH) agreed on inclusion/exclusion decisions in 93.5% of cases (Cohen’s κ = 0.63), reflecting substantial agreement beyond chance. Meanwhile, during full-text screening, agreement between Reviewer A and Reviewer B was perfect, with 100% concordance (Cohen’s κ = 1.00).

### 2.3. Quality Assessment

The quality of the included studies was assessed using the Newcastle–Ottawa Scale (NOS). The tool was modified for case–control and cohort studies and adapted for cross-sectional designs. Each study underwent detailed evaluation and was rated as having high, moderate, or low quality based on NOS criteria, with scores presented in Supplementary Table S2. For studies with unclear design, the cross-sectional NOS was applied. All assessments were independently conducted by ZMH and subsequently reviewed by CMNCMN. Importantly, quality ratings were not used as criteria for study exclusion or to differentially weight effect sizes in the meta-analysis; instead, all eligible studies were included, and NOS scores were used to contextualize the robustness of the evidence base in the narrative synthesis. NOS scores were not incorporated into sensitivity analyses or meta-regression because the distribution of scores was relatively narrow, with most studies rated as moderate-to-high quality, and the limited number of studies within key subgroups precluded stable quality-stratified analyses.

### 2.4. Data Extraction and Meta-Analysis

For data extraction, a standardized checklist was developed, which included the following variables: first author’s name, year of publication, country/region, study design, sample size, point prevalence, and type of sleep disorder, mean age of participants, diagnostic methods, glymphatic assessment, and ePVS rating/scale/location. When studies reported multiple sleep disorder categories within the same cohort (e.g., insomnia, obstructive sleep apnea, parasomnias), prevalence was extracted at the study level as the proportion of participants with any sleep disorder, as reported by the original authors. To avoid double-counting individuals with comorbid sleep disorders, disorder-specific prevalence estimates were not summed. If an overall prevalence of any sleep disorder was not explicitly provided, a single prevalence estimate corresponding to the primary sleep outcome defined by the study was extracted. Studies reporting only disorder-specific prevalences without sufficient information to derive an overall “any sleep disorder” prevalence were handled conservatively and not aggregated across categories. In addition, across included studies, glymphatic-related measures were not acquired under standardized physiological states. Most human imaging studies assessed structural and functional proxies during wakeful resting-state MRI sessions, whereas a minority of dynamic imaging studies approximated sleep or altered arousal conditions. Due to inconsistent reporting and limited study numbers, stratification by physiological state at acquisition (sleep vs. wake) was not feasible.

Extracted data were entered into the IBM Statistical Package for the Social Sciences (SPSS) for Windows, Version 29.0 (IBM Corp., Armonk, NY, USA) with the meta-analysis extension package. Prevalence data were extracted as proportions and transformed using the logit function to stabilize variance, particularly for studies with very low or very high prevalence (see formulas 1 to 4 below).

(1) $$p\;\left(\mathrm{proportion}\;\mathrm{or}\;\mathrm{point}\;\mathrm{prevalence}\right)=\frac{\mathrm{Number}\;\mathrm{of}\;\mathrm{cases}\;\left(x\right)\;}{\mathrm{Total}\;\mathrm{sample}\;\mathrm{size}\;\left(N\right)}$$

(2) $$\mathrm{Corrected}\;\mathrm{proportion}\;\left(p_{\mathrm{Corr}}\right)=\frac{x+0.5}{N+1}$$

(3) $$\mathrm{Logit}\;\mathrm{transfomation},\;\mathrm{logit}\;\left(p\right)=\ln(\frac{p_{\mathrm{Corr}}}{1-p_{\mathrm{Corr}}})$$

(4) $$\mathrm{Variance}\;\mathrm{of}\;\mathrm{logit},\;\mathrm{Var}\left(\mathrm{logit}\;\left(p\right)\right)=\frac{1}{x+0.5}+\frac{1}{\left(N-x\right)+0.5}$$

Pooled effect sizes were estimated under a random-effects model with restricted maximum likelihood (REML), incorporating both within- and between-study variance. Summary estimates were reported as logit-transformed effect sizes with 95% confidence intervals (CIs) and subsequently back-transformed to proportions (%) with corresponding 95% CIs for interpretability.

$$\mathrm{logit}\;\mathrm{back}\;\mathrm{transformed},\;p=\frac{e^{\mathrm{logit}}}{1+e^{\mathrm{logit}}}$$

$$\mathrm{Lower}\;\mathrm{CI}=\frac{e^{\mathrm{logit}\left(\mathrm{low}\right)}}{1+e^{\mathrm{logit}\left(\mathrm{low}\right)}}$$

$$\mathrm{Upper}\;\mathrm{CI}=\frac{e^{\mathrm{logit}\left(\mathrm{high}\right)}}{1+e^{\mathrm{logit}\left(\mathrm{high}\right)}}$$

Between-study heterogeneity was assessed using Cochran’s Q test and quantified with the I^2^ statistic, which estimates the proportion of variability due to true heterogeneity rather than chance. Tau^2^ values were also calculated as an absolute measure of between-study variance. A fixed-effects model was applied when heterogeneity was negligible; otherwise, random-effects estimates were reported. To investigate potential sources of heterogeneity, subgroup analyses were conducted according to study region (Asia, Europe, North America, South America) and study design (case–control, cohort, cross-sectional). Between-subgroup differences were formally tested using Q statistics for subgroup homogeneity. Additionally, meta-regression analyses were performed on study-level moderators, including mean age, sample size, and study year. Publication bias and small-study effects were assessed visually through funnel plot asymmetry and formally tested using Egger’s regression test. A two-tailed *p* < 0.05 was considered statistically significant.

## 3. Results

### 3.1. Study Selection and Quality

The initial search yielded a total of 686 records, of which 52 duplicates were removed. After title and abstract screening, 45 studies were assessed in full text, and 13 studies ultimately fulfilled the eligibility criteria and were included in both the qualitative synthesis and meta-analysis. However, during the process of data extraction and preparation of the quantitative synthesis, six additional eligible studies were identified through reference list screening and citation tracking. These were subsequently included, bringing the total number of studies included in the meta-analysis to 19. Moreover, during screening, no eligible studies were excluded solely based on language if data extraction was feasible from the English abstract or translated full text. The study selection process is summarized in Figure 1, which presents the PRISMA 2020 flow diagram.

The methodological quality of the included studies was assessed using the NOS, adapted for case–control, cohort, and cross-sectional designs (see Supplementary Table S2). Scores across the three domains of selection, comparability, and outcome ranged from 6 to 8 out of a maximum of 9. Among the cohort and case–control studies, most achieved scores of 8/9, reflecting representative sampling, adequate ascertainment of exposure, and robust outcome assessment, although some studies lost points due to lack of external controls or incomplete reporting of comparability. The cross-sectional studies demonstrated slightly more variability, with scores ranging from 6/9 to 8/9. Studies such as Du et al. [22], Gui et al. [23], and Opel et al. [24] were rated 6/9, primarily due to limitations in addressing non-respondents and controlling for confounding factors, whereas others, such as Zhao et al. [25,26], scored 8/9, indicating high methodological rigor with validated outcome measures and appropriate statistical testing. Overall, based on NOS thresholds, most studies were rated as high quality (scores 7–9), with a minority falling in the moderate-quality range (scores 6/9). While no single study met the NOS criteria for high risk of bias, the overall body of evidence should be interpreted with caution, given the predominance of small, cross-sectional studies and between-study heterogeneity. These factors increase the likelihood of residual confounding and selective reporting, which may inflate prevalence estimates despite acceptable individual study quality.

### 3.2. Study Characteristics

The nineteen (19) studies included in the review were published between 2018 and 2025 and were conducted across Asia, Europe, and the Americas (Supplementary Table S3). The majority adopted cross-sectional case–control designs, while three employed longitudinal or prospective approaches. The total sample size was 2798, ranging from 36 to 1076 participants, with a mean of 147.3 (SD = 228.41). The populations under study were diverse and included community-dwelling adults, patients with cerebral small vessel disease (CSVD), individuals with PD, narcolepsy type 1 (NT1), autism spectrum disorder (ASD), succinic semialdehyde dehydrogenase deficiency (SSADHD), Down syndrome, and cohorts with long COVID or post-Omicron infection. The mean participant age across studies ranged from early adulthood to older age, reflecting the heterogeneity of the populations examined.

Based on Supplementary Table S3, sleep disorders were variably defined and diagnosed. Insomnia disorders (i.e., poor sleep quality, sleep fragmentation, and sleep disturbance) were the most commonly investigated condition, followed by sleep-disordered breathing (i.e., OSA and periodic limb movements in sleep [PLMS]), REM-related parasomnias (i.e., isolated REM sleep behavior disorder [iRBD]), excessive daytime sleepiness (EDS), narcolepsy, and sleep-related movement disorder (i.e., restless leg syndrome). Occurrence and/or prevalence values reported in Supplementary Table S3 reflect point occurrence/prevalence estimates, based on the proportion of participants with the outcome at the time of assessment. Diagnostic methods included objective PSG, applied according to American Academy of Sleep Medicine (AASM) or International Classification of Sleep Disorders, Third Edition (ICSD-3) criteria, as well as validated self- or caregiver-report instruments such as the Pittsburgh Sleep Quality Index (PSQI), Epworth Sleepiness Scale (ESS), Insomnia Severity Index (ISI), and the Children’s Sleep Habits Questionnaire (CSHQ). In some studies, clinical diagnostic standards such as the ICSD-3 were employed to confirm conditions such as NT1.

Assessment of glymphatic-related dysfunction relied on a range of neuroimaging and biomarker techniques. Several studies employed MRI-based quantification of ePVS, either in the basal ganglia, centrum semiovale, or global white matter, with some also including WMHs burden as a proxy of impaired clearance. The DTI-ALPS was used in two recent studies, while ultrafast fMRI (MR-encephalography) was employed to capture brain pulsation dynamics. One study examined gamma aminobutyric acid (GABA) levels using magnetic resonance spectroscopy (MRS) in conjunction with ePVS burden. Collectively, these studies provide converging but methodologically diverse evidence on the association between sleep disturbances and glymphatic dysfunction. As all studies reported glymphatic dysfunction using continuous measures (e.g., ePVS burden, DTI-ALPS index, WMHs volume, CSF/PET biomarkers), no dichotomization was applied. Prevalence estimates of sleep disorders were extracted as reported for the entire study populations, without stratification by glymphatic measure thresholds. A full summary of study characteristics is presented in Supplementary Table S3.

### 3.3. Meta-Analysis of Pooled Prevalence

**Figure 2:**
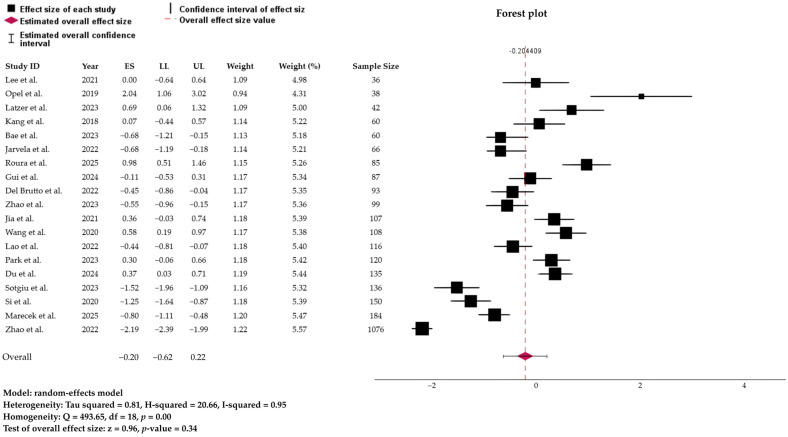
Forest plot and distribution of true effect, representing the global prevalence of sleep disorders linked to the glymphatic system based on the Random Effect Model. ES, effect size; LL/UL, lower and upper limit of 95% confidence interval. References: Du et al. [22]; Gui et al. [23]; Opel et al. [24]; Zhao et al. [25,26]; Latzer et al. [27]; Kang et al. [28]; Wang et al. [29]; Jia et al. [30]; Del Brutto et al. [31]; Jarvela et al. [32]; Lao et al. [33]; Sotgiu et al. [34]; Park et al. [35]; Roura et al. [36]; Marecek et al. [37]; Bae et al. [38]; Si et al. [39]; and Lee et al. [40]. The size of each square is proportional to the inverse-variance weight of the study in the random-effects model.

A random-effects meta-analysis estimated the pooled prevalence of sleep disorders linked to glymphatic-related dysfunction, estimated using a random-effect model weighted by effect size, was at 44.92% (95% CI: 34.89% to 55.35%). However, the prediction interval ranged widely from 10.3% to 85.2%, reflecting substantial between-study heterogeneity (Figure 2). The pooled estimates represent the occurrence of at least one sleep disorder per participant and do not reflect mutually exclusive diagnostic categories. The test of homogeneity confirmed significant variability across studies (Q = 493.65, df = 18, *p* < 0.001). Consistently, heterogeneity indices were high (τ^2^ = 0.81; I^2^ = 95.2%; H^2^ = 20.66), indicating that nearly all of the observed variance in prevalence estimates was due to real differences across studies rather than chance. Given the very high heterogeneity (I^2^ = 95.2%) and the wide prediction interval (10.3–85.2%), the global pooled occurrence should be interpreted as a descriptive summary of the overall burden of sleep disorders across heterogeneous populations with glymphatic-related impairment rather than as a precise or generalizable prevalence estimate.

**Figure 3:**
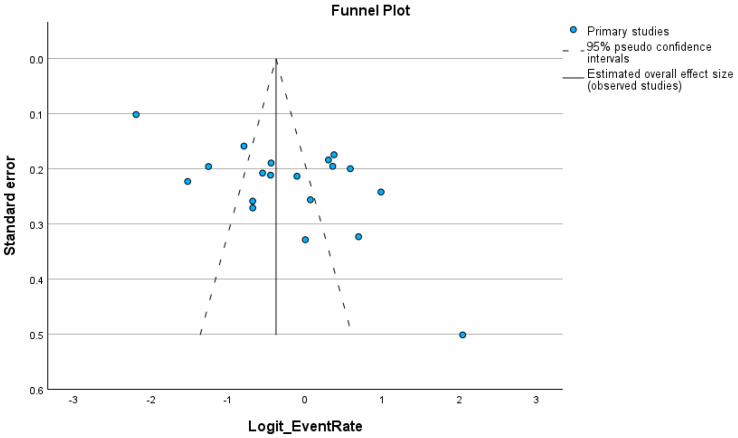
Funnel plot representing distribution bias in the reviewed studies.

Visual inspection of the funnel plot (Figure 3) revealed asymmetry, with smaller studies reporting higher prevalence estimates clustering on the right side of the graph, suggesting small-study effects. This was statistically supported by Egger’s regression intercept (B_0_ = 7.49, 95% CI: 2.67–12.32; t = 3.28; *p* = 0.0043). In the absence of publication bias, studies should be symmetrically distributed around the pooled effect within the triangular confidence limits. Instead, the observed asymmetry indicates potential publication bias. Using the trim-and-fill method, two studies were imputed on the left side of the funnel, reducing the pooled prevalence from 44.92% to 40.64% (95% CI, 30.24–51.92%; *p* = 0.104), indicating that the bias-adjusted estimate is somewhat lower, but the confidence intervals overlap, so the change is not dramatic. Sensitivity analyses conducted sequentially, excluding individual studies, demonstrated that no single study exerted a disproportionate influence on the overall pooled estimate. The leave-one-out procedure consistently yielded prevalence estimates within the 95% confidence interval of the main analysis, confirming the stability and robustness of the findings.

### 3.4. Subgroup Analysis: Prevalence by Regions, Study Designs, and Sleep Disorder Categories

**Figure 4:**
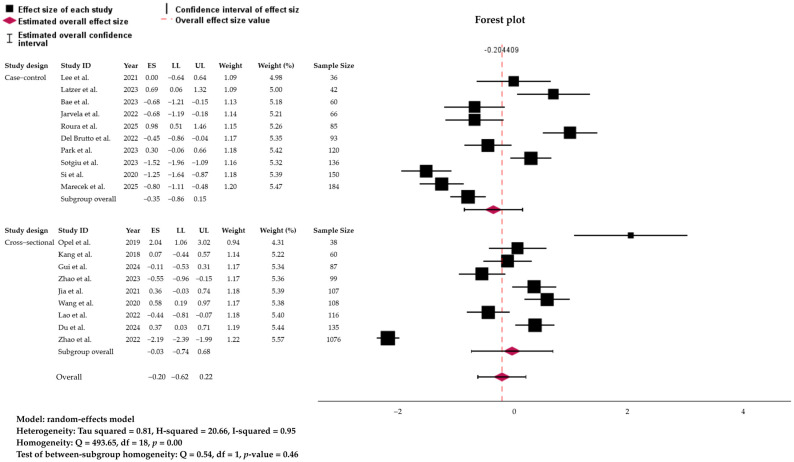
Forest plot and distribution of true effect, representing the subgroup analysis of the global prevalence of sleep disorders linked to the glymphatic-related activity by the study’s design. Analysis is based on the Random Effect. ES, effect size; LL/UL, lower and upper limit of 95% confidence interval. References: Du et al. [22]; Gui et al. [23]; Opel et al. [24]; Zhao et al. [25,26]; Latzer et al. [27]; Kang et al. [28]; Wang et al. [29]; Jia et al. [30]; Del Brutto et al. [31]; Jarvela et al. [32]; Lao et al. [33]; Sotgiu et al. [34]; Park et al. [35]; Roura et al. [36]; Marecek et al. [37]; Bae et al. [38]; Si et al. [39]; and Lee et al. [40]. The size of each square is proportional to the inverse-variance weight of the study in the random-effects model.

**Figure 5:**
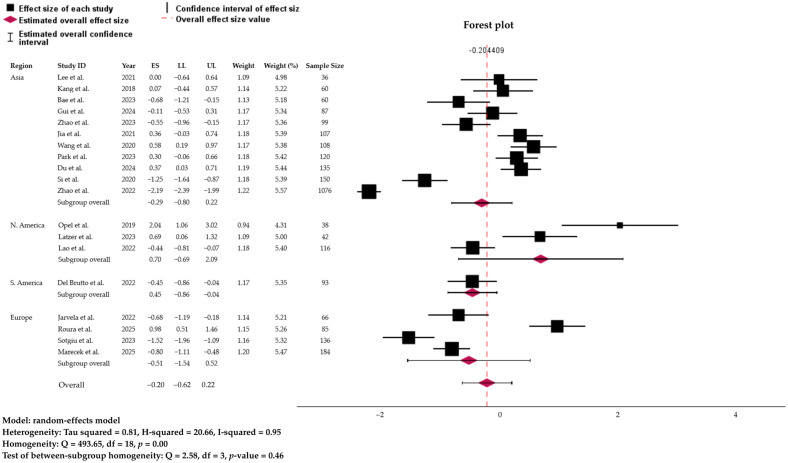
Forest plot and distribution of true effect, representing the subgroup analysis of the global prevalence of sleep disorders linked to the glymphatic-related activity by the study’s regions. Analysis is based on the Random Effect. ES, effect size; LL/UL, lower and upper limit of 95% confidence interval. References: Du et al. [22]; Gui et al. [23]; Opel et al. [24]; Zhao et al. [25,26]; Latzer et al. [27]; Kang et al. [28]; Wang et al. [29]; Jia et al. [30]; Del Brutto et al. [31]; Jarvela et al. [32]; Lao et al. [33]; Sotgiu et al. [34]; Park et al. [35]; Roura et al. [36]; Marecek et al. [37]; Bae et al. [38]; Si et al. [39]; and Lee et al. [40]. The size of each square is proportional to the inverse-variance weight of the study in the random-effects model.

Table 2 summarizes the pooled prevalence of sleep disorders stratified by study type, geographical region, and disorder subtype. Across study designs, cross-sectional studies (9 studies; *n* = 972) yielded a pooled prevalence of 41.3% (95% CI: 29.7–53.7), while case–control studies (10 studies; *n* = 1826) showed a slightly higher prevalence of 49.3% (95% CI: 32.3–66.4) (Figure 4). Regional analyses revealed considerable variation. Studies conducted in North America demonstrated the highest prevalence (66.8%, 95% CI: 33.4–89.1), although based on only three studies with small sample sizes. Asia, representing the largest regional sample (11 studies; *n* = 2038), showed a prevalence of 42.8% (95% CI: 31.0–55.5), comparable to Europe (37.5%, 95% CI: 17.7–62.7) and South America (38.9%, 95% CI: 29.8–49.0) (Figure 5).

**Figure 6:**
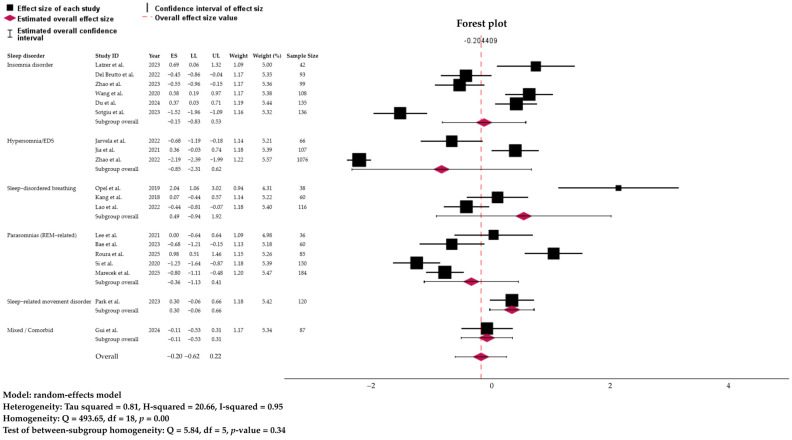
Forest plot and distribution of true effect, representing the subgroup analysis of the global prevalence of sleep disorders linked to the glymphatic-related activity by study’s sleep disorder categories. Analysis is based on the Random Effect. ES, effect size; LL/UL, lower and upper limit of 95% confidence interval. References: Du et al. [22]; Gui et al. [23]; Opel et al. [24]; Zhao et al. [25,26]; Latzer et al. [27]; Kang et al. [28]; Wang et al. [29]; Jia et al. [30]; Del Brutto et al. [31]; Jarvela et al. [32]; Lao et al. [33]; Sotgiu et al. [34]; Park et al. [35]; Roura et al. [36]; Marecek et al. [37]; Bae et al. [38]; Si et al. [39]; and Lee et al. [40]. The size of each square is proportional to the inverse-variance weight of the study in the random-effects model.

When categorized by disorder type, sleep-related movement disorders exhibited the highest pooled prevalence at 57.4% (95% CI: 48.5–65.9), followed by mixed/comorbid disorders (47.2%, 95% CI: 37.0–57.3) and insomnia disorders (46.3%, 95% CI: 30.3–62.9). In contrast, hypersomnia/EDS had the lowest prevalence (29.9%, 95% CI: 9.0–65.0). Sleep-disordered breathing and REM-related parasomnias were intermediate, with pooled prevalence estimates of 37.9% (95% CI: 28.0–87.2) and 41.0% (95% CI: 24.3–60.1), respectively (Figure 6). Marked heterogeneity was observed across nearly all subgroups (I^2^ > 90%), reflecting substantial variability in study populations, diagnostic criteria, and methodological approaches. Egger’s test values, although variable, further suggested potential small-study effects. Notably, sleep-related movement disorders and insomnia emerged as the most prevalent categories worldwide, whereas hypersomnia was the least common.

### 3.5. Meta-Regression on Study-Level Moderators

**Figure 7:**
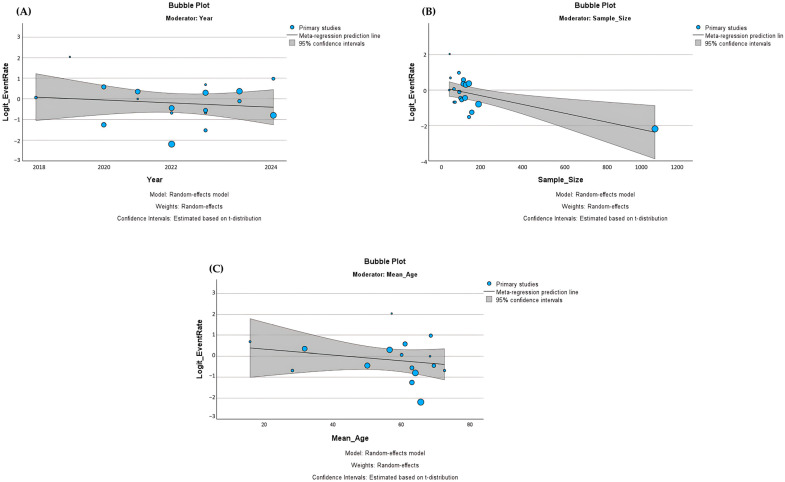
Bubble plots of meta-regression analyses. Meta-regression was conducted to explore potential moderators of heterogeneity in prevalence estimates. (**A**) sample size, (**B**) year of paper publication, and (**C**) mean age were examined as moderators. Each circle represents primary study, with bubble size proportional to study weight. The solid line indicates the meta-regression prediction line, and the shaded area represents the 95% confidence interval. No significant associations were observed between prevalence and year of publication, sample size, or mean age. Circle size represent study weight.

Meta-regression analyses were conducted to explore potential moderators of the observed heterogeneity using study-level moderators, including publication year, sample size, mean age, and sleep disorder categories (Table 3). None of the moderators significantly influenced the pooled prevalence of sleep disorders. Publication year showed no effect (β = −0.030, 95% CI: −0.389 to 0.329, *p* = 0.86), indicating no temporal trend in reported prevalence estimates (Figure 7A). Sample size also did not significantly moderate prevalence (β = −0.002, 95% CI: −0.005 to −0.00007, *p* = 0.44), although it explained 14.2% of the between-study variance (R^2^ = 14.2%) (Figure 7B). Mean age demonstrated a non-significant negative association (β = −0.030, 95% CI: −0.065 to 0.014, *p* = 0.19), accounting for only 3.1% of the observed variance (Figure 7C). Overall, the meta-regression results suggest that heterogeneity (I^2^ > 90% across models) remains largely unexplained by these study-level characteristics.

## 4. Discussion

This systematic review and meta-analysis synthesized evidence from 19 studies involving 2798 individuals to quantify the prevalence (or occurrence) of sleep disorders in populations with impaired glymphatic function. The pooled prevalence was 44.92% (95% CI: 34.89 to 55.35%), with substantial heterogeneity (I^2^ = 95.2%). These findings extend earlier reviews that described the bidirectional role of sleep in glymphatic-related activities by offering the first pooled estimate across diverse populations and diagnostic modalities [41]. Our results suggest that nearly one in two individuals with evidence of glymphatic-related impairment also experiences a sleep disorder. This prevalence is considerably higher than estimates in the general population, where chronic insomnia and OSA typically affect 10–30% of adults [42,43]. For example, large-scale surveys in Europe, Australia, and North America report chronic insomnia prevalence of ~15% and OSA prevalence of ~10% [44,45,46]. The higher prevalence in our synthesis underscores the tight interdependence between sleep regulation and glymphatic-related activities. Comparable findings were observed in specific cohorts: Zhao et al. [25] reported poorer sleep quality among older adults with CSVD and ePVS, while Del Brutto et al. [31] and Du et al. [22] highlighted disproportionately high insomnia rates in post-COVID populations. Our results suggest that newer studies, particularly those conducted in post-COVID-19 populations, reported a higher prevalence of sleep disturbances. However, this should be interpreted as a correlative observation rather than a causal effect, given the cross-sectional and case–control designs of the included studies. While post-infectious neuroinflammation and vascular changes may plausibly contribute to both sleep impairment and glymphatic-related dysfunction, longitudinal studies are needed to establish temporal sequence and causality.

In addition, the very high heterogeneity (I^2^ = 95.2%) is comparable to other sleep epidemiology meta-analyses, such as global prevalence reviews of insomnia and sleep apnea, where I^2^ often exceeds 90% due to differences in populations, instruments, and diagnostic thresholds [47,48]. Moreover, the consistently high heterogeneity (I^2^ > 90%) indicates that the prevalence of sleep disorders in populations with glymphatic-related impairment varies widely across populations, clinical contexts, and measurement modalities. Consequently, the global pooled estimate should be viewed as a high-level descriptive benchmark rather than a definitive or universally applicable prevalence. In this context, the prediction interval is arguably more informative than the pooled point estimate, as it captures the plausible range of true prevalence across real-world settings. The substantial dispersion observed underscores the context-specific nature of the sleep–glymphatic relationship and cautions against overgeneralization of a single summary value.

The proxies of glymphatic-related dysfunction included in this meta-analysis capture distinct but mechanistically linked dimensions of impaired brain clearance. Functional measures such as DTI-ALPS, ultrafast fMRI, and CSF/PET clearance indices more directly index perivascular fluid transport and solute exchange, whereas ePVS and WMHs reflect downstream structural and tissue-level consequences of chronic perivascular and microvascular dysfunction. These modalities, therefore, lie along a biological continuum rather than representing interchangeable biomarkers. Our pooled estimate should thus be interpreted as reflecting the co-occurrence of sleep disorders with impaired brain clearance–related processes broadly construed, rather than as a precise quantitative estimate of glymphatic transport impairment.

In addition, subgroup analysis showed no significant differences between cross-sectional and case–control studies (Q = 0.094, *p* = 0.760), in line with prior systematic reviews where study design per se did not drive heterogeneity in sleep prevalence estimates [49]. However, the wide confidence intervals among cross-sectional studies in our review highlight the instability of small-sample prevalence estimates, a problem also recognized in general sleep epidemiology. Meta-regression findings deepen this picture. Despite not being significant, the negative trends between sample size and prevalence mirror patterns observed in prior meta-analyses of psychiatric disorders, where smaller studies frequently report inflated prevalence due to selective sampling and clinic-based recruitment [50,51]. On the other hand, the negative trends between year of publication and prevalence were comparable with some older meta-analyses of sleep disorders in the general population, which tended to show stable or even declining prevalence [52].

Moreover, although we considered conducting subgroup analyses by glymphatic-related assessment type (e.g., ePVS, WMHs, DTI-ALPS, or CSF biomarkers), this was not feasible due to the limited number of studies available within each modality. For most measures, only one to three studies met the inclusion criteria, which precluded meaningful stratification and would have resulted in underpowered and unstable estimates. We therefore opted to pool these markers under the broader conceptual framework of glymphatic-related dysfunction while carefully acknowledging the potential heterogeneity introduced by diverse operational definitions. As the field advances and more studies employing standardized methodologies emerge, future meta-analyses will be better positioned to explore modality-specific effects. In addition, our results suggest that in the specific context of glymphatic-related dysfunction, newer methods (e.g., DTI-ALPS, ultrafast fMRI) and contextual factors (e.g., post-COVID-19 increases in sleep disturbances) have raised indirect detection rates in recent years. This is consistent with emerging work linking COVID-19-related neuroinflammation to both sleep impairment and glymphatic pathology [53].

However, the interpretation of these estimates must be tempered by evidence of bias. The funnel plot demonstrated clear asymmetry, with Egger’s regression confirming small-study effects. Smaller studies reported disproportionately higher prevalence, while larger cohorts clustered nearer to the null. This pattern suggests possible publication bias or selective reporting of positive findings. After trim-and-fill adjustment, the prevalence was reduced to 40.64% (95% CI, 30.24–51.92%), though it remained statistically nonsignificant (*p* = 0.104), which is likely a more conservative and realistic reflection of the true burden. While this adjusted estimate is lower, it still exceeds background prevalence in the general population, reinforcing a meaningful relationship between sleep disorders and impaired glymphatic-related function. Although our findings suggest a consistent association between sleep disorders and glymphatic-related dysfunction, evidence of small-study effects and potential publication bias limits the robustness of clinical and preventive inferences. The current literature is dominated by relatively small, single-center studies, often from a limited number of geographic regions, which may inflate effect estimates and constrain external validity. Accordingly, any clinical or preventive implications should be viewed as hypothesis-generating rather than prescriptive. Extrapolation to underrepresented regions and populations should be undertaken with caution, and confirmation in large, multicenter, and geographically diverse cohorts using harmonized protocols is required before firm recommendations can be made.

Moreover, mechanistic evidence supports these epidemiological findings. Aquaporin-4 (AQP4) water channels on astrocytic end feet regulate perivascular CSF–ISF exchange, and their mislocalization impairs both Aβ clearance and sleep–wake regulation [54,55]. Human imaging studies further link disrupted AQP4 polarization with reduced sleep efficiency and cognitive decline [56]. Vascular stiffness, another driver of glymphatic dysfunction, reduces perivascular pulsatility and clearance efficiency [57]. Consistent with this, studies included in our review reported associations between ePVS, WMHs, and poor sleep quality [31,33]. In addition, neuroinflammation represents a third mechanistic pathway: post-COVID cohorts demonstrated high insomnia prevalence [22,31], aligning with evidence that systemic inflammation impairs glymphatic transport and destabilizes sleep. Interestingly, beyond the context of COVID-19, there is a converging mechanistic rationale linking neuroinflammation to impaired glymphatic function that helps explain the associations observed across diverse clinical conditions. Systemic or CNS-directed inflammatory responses activate microglia and astrocytes and increase pro-inflammatory cytokines (e.g., IL-1β, TNF-α), which can alter astrocyte end-foot morphology and disrupt the perivascular localization of AQP4 [58]. Loss or mislocalization of AQP4 impairs water flux across astrocytic end feet and reduces perivascular CSF–ISF exchange. In parallel, inflammation and endothelial dysfunction promote blood–brain barrier (BBB) disruption and reduce arterial/arteriolar pulsatility, a key driver of perivascular solute transport [58], thereby further diminishing clearance.

In addition, chronic inflammation also promotes extracellular matrix remodeling and perivascular fibrosis, which may narrow perivascular spaces and slow convective flow [59,60]. Together, these processes can produce a feed-forward cycle whereby impaired clearance increases accumulation of neurotoxic proteins and debris, which in turn amplifies local inflammation and neuronal dysfunction. These mechanisms (microglial/astrocytic activation, AQP4 dysfunction, BBB and vascular impairment, perivascular structural change) span a range of pathologies, including aging, cerebrovascular disease, neurodegenerative disorders, chronic infection, and autoimmune encephalopathies, and provide biologically plausible pathways linking inflammatory states with the glymphatic alterations summarized in our meta-analysis. Finally, the observed association between sleep disorders and glymphatic-related dysfunction should be interpreted within a bidirectional framework. Experimental and clinical evidence indicates that sleep disruption can directly impair perivascular fluid transport and solute clearance, whereas chronic glymphatic and neurovascular dysfunction may, in turn, exacerbate sleep–wake disturbances via neuroinflammatory signaling, network disconnection, and altered arousal regulation [61,62]. As most included studies were cross-sectional, temporal precedence cannot be established, and reverse causality remains plausible. Accordingly, our findings reflect co-occurrence and coupling between sleep pathology and impaired brain clearance–related processes rather than a unidirectional causal effect. Overall, these mechanisms support the concept of a vicious cycle in which sleep disruption and impaired glymphatic clearance reinforce one another, accelerating toxic protein accumulation and risk of neurodegeneration.

### Methodological Strengths, Limitations, and Future Directions

This review’s strengths include comprehensive population coverage, use of diverse imaging and biomarker modalities, and rigorous risk-of-bias assessment. Sensitivity analyses confirmed the stability of findings. Nonetheless, several limitations merit caution. First, heterogeneity remained extremely high with a wide prediction interval (10.3% to 85.2%), despite subgroup and meta-regression analyses, reflecting differences in study design, populations, and diagnostic tools. The extremely high heterogeneity observed across analyses (I^2^ consistently > 90%) limits the interpretability and clinical generalizability of the global pooled prevalence. While random-effects modeling accounts for between-study variance, such levels of heterogeneity indicate that prevalence estimates are strongly context-dependent and influenced by population characteristics, diagnostic criteria for sleep disorders, and the modality used to index glymphatic-related dysfunction. Accordingly, the pooled estimate should be interpreted as descriptive and hypothesis-generating rather than as a precise epidemiological parameter. Although subgroup analyses were conducted, modality-specific meta-analyses were not feasible due to the limited number of studies per biomarker class. As the evidence base expands, future syntheses should prioritize modality-specific and disease-specific pooling to derive more clinically actionable estimates.

Another important source of heterogeneity that we could not formally examine is variation in sleep disorder ascertainment across studies. Included studies employed a mix of objective polysomnography-based diagnoses (according to AASM/ICSD-3 criteria) and questionnaire-based or self-/caregiver-reported instruments (e.g., PSQI, ISI, ESS, CSHQ). Questionnaire-based measures capture perceived sleep disturbance and symptom severity rather than definitive clinical diagnoses and may be subject to reporting bias and misclassification. Differences in diagnostic modality may therefore contribute to the substantial between-study heterogeneity observed. Although subgroup or meta-regression analyses by diagnostic method would be informative, the limited number of studies within each category precluded statistically robust stratification. Additionally, the handling of overlapping and comorbid sleep disorders represents another potential source of measurement bias. Although we avoided double-counting by extracting prevalence at the study level and did not sum disorder-specific prevalences, some primary studies did not fully report comorbidity structures or provide mutually exclusive diagnostic categories. As a result, the pooled estimates reflect the occurrence of “any sleep disorder” and may mask differential patterns across specific disorders or combinations of disorders. As the evidence base expands, future primary studies and meta-analyses should report mutually exclusive diagnostic categories and comorbidity profiles, and stratify prevalence estimates by diagnostic modality, prioritizing PSG-confirmed sleep disorders, to improve precision, comparability, and clinical interpretability.

Furthermore, a major limitation of this meta-analysis is the conceptual heterogeneity of the glymphatic-related proxies included. Pooling functional transport indices (DTI-ALPS, ultrafast fMRI, CSF/PET clearance markers) with structural correlates (ePVS, WMHs) introduces biological non-equivalence and may obscure modality-specific relationships with sleep disorders. Structural markers likely reflect cumulative downstream consequences of impaired clearance rather than glymphatic transport itself. This heterogeneity may partially explain the very high between-study variance observed. The pooled estimate should therefore be interpreted as an exploratory summary of converging evidence linking sleep disorders with impaired brain clearance–related processes, rather than a definitive measure of glymphatic dysfunction. As the field matures, future meta-analyses should stratify by biomarker class and prioritize direct functional measures of perivascular fluid transport. Apart from that, the glymphatic transport is strongly state-dependent, with substantially greater CSF–interstitial fluid exchange during sleep and anesthesia than during wakefulness. However, the included studies varied in physiological state at the time of measurement, and most human studies obtained glymphatic-related proxies during wakeful resting-state MRI. This variability likely contributes to between-study heterogeneity and may attenuate observed associations, particularly when pooling state-dependent functional measures with trait-like structural markers such as ePVS and WMHs. The inability to standardize or model sleep–wake state, circadian timing, or recent sleep history represents an important limitation. Future studies should harmonize acquisition protocols and explicitly report physiological state to improve comparability and biological interpretability.

Additionally, although individual studies were largely rated as moderate-to-high quality on NOS, the evidence base as a whole is limited by publication bias and small-study effects likely inflated prevalence, as indicated by funnel plot asymmetry and trim-and-fill correction. These factors collectively raise the risk of overestimating prevalence and reduce certainty in the pooled estimate. NOS scores were not incorporated into sensitivity analyses or meta-regression. This limits the formal evaluation of the influence of methodological quality on pooled prevalence estimates. The relatively narrow distribution of NOS scores and the small number of studies within several subgroups constrained the feasibility and interpretability of quality-stratified analyses. As the evidence base grows, future meta-analyses should incorporate risk-of-bias–informed sensitivity analyses or meta-regression to better quantify the impact of study quality on pooled estimates. Finally, most included studies were cross-sectional, limiting causal inference and raising the possibility that sleep disturbance and glymphatic-related impairment are mutually reinforcing rather than unidirectional.

Finally, the predominantly cross-sectional nature of the included studies precludes causal inference and does not allow disentanglement of directionality between sleep disorders and glymphatic-related dysfunction. Reverse causality is plausible, whereby sleep disruption may directly impair glymphatic transport, while impaired clearance and neurovascular injury may reciprocally worsen sleep–wake regulation. Longitudinal cohort studies and interventional trials incorporating pre–post glymphatic imaging following sleep manipulation or treatment (e.g., CPAP for obstructive sleep apnea, CBT-I for insomnia) are needed to clarify causal pathways. Evidence of small-study effects suggests potential publication and reporting bias, which may overestimate pooled prevalence and associations. Moreover, the geographic distribution of included studies was uneven, with underrepresentation of low- and middle-income regions, limiting global generalizability. These factors constrain the robustness of clinical or preventive recommendations and highlight the need for larger, preregistered, multicenter studies across diverse populations to validate the observed associations.

However, these findings carry important implications for public health and clinical practice. Sleep disorders already contribute substantially to global disability and economic burden; their high prevalence in populations with impaired glymphatic-related function suggests that sleep disorders may be associated with alterations in glymphatic activity and could potentially serve as indirect indicators of glymphatic health. It is also important to consider the possibility of reverse causality. Rather than glymphatic dysfunction predisposing to sleep disorders, chronic sleep disturbances themselves may impair glymphatic clearance by disrupting slow-wave sleep, altering cerebrovascular dynamics, and promoting neuroinflammation [63,64]. This raises the likelihood of a bidirectional relationship, in which sleep disorders and glymphatic impairment reinforce one another over time. Future longitudinal studies will be essential to clarify the temporal sequence and causal pathways.

Integrating sleep assessments into neurodegenerative risk prediction models could improve early detection of individuals at risk for AD, PD, and cerebrovascular disease (including CSVD) [65]. From a translational perspective, advancing this field will require the adoption of standardized diagnostic frameworks. For sleep disorders, existing consensus-based systems such as the ICSD-3 and AASM guidelines provide validated diagnostic criteria that should be consistently applied. In contrast, no equivalent consensus yet exists for glymphatic assessment. Current studies use heterogeneous biomarkers, ranging from MRI-based ePVS and WMHs scoring to DTI-ALPS, ultrafast fMRI, and CSF/PET markers, each with different thresholds, scoring systems, and reproducibility [15,66]. We propose that consensus guidelines should prioritize: (i) harmonized imaging protocols and quantitative scoring methods, (ii) standardized thresholds for impairment, and (iii) integration of multimodal biomarkers into cross-cohort validation frameworks. The development of such consensus standards will be essential for comparability across studies, for pooling data in meta-analyses, and ultimately for clinical translation. Moreover, interventional trials are also warranted to test whether therapies that promote slow-wave sleep, restore AQP4 polarity, or improve vascular pulsatility can mitigate glymphatic impairment and downstream neurodegeneration.

At the policy level, these findings support the inclusion of sleep screening in preventive neurology and public health programs. Simple validated tools such as the PSQI or ESS could be implemented in community settings to identify individuals at risk of glymphatic dysfunction, particularly in high-risk groups such as those with CSVD, long COVID, or prodromal neurodegenerative disease. Preventive strategies promoting healthy sleep through public health campaigns, workplace wellness programs, and clinical guidelines may represent one of the most cost-effective approaches to preserving brain clearance capacity and reducing the burden of dementia and stroke globally. Finally, future research should prioritize longitudinal and interventional studies to clarify causality, including whether sleep disruption initiates glymphatic dysfunction or vice versa. Multimodal imaging combined with CSF biomarkers and objective sleep studies will be crucial to establishing mechanistic links. Additionally, global epidemiological studies are needed to reduce regional bias and assess population disparities. Finally, contextual influences such as COVID-19-related neuroinflammation should be systematically evaluated as potential modifiers of the sleep–glymphatic relationship.

## 5. Conclusions

This systematic review and meta-analysis demonstrate that sleep disorders are highly prevalent in individuals with impaired glymphatic-related function, with a pooled prevalence of 44.92% (95% CI: 34.89 to 55.35%) across 19 studies involving 2798 individuals. Even after accounting for publication bias, the adjusted prevalence remained 40.64%, substantially higher than estimates in the general population. Although heterogeneity and small-study effects temper the precision of these estimates, the consistency of the association across clinical and community cohorts, including patients with CSVD, PD, ASD, and post-COVID populations, highlights a robust and clinically meaningful relationship. Beyond prevalence, our findings reinforce a growing mechanistic framework: sleep and glymphatic-related function are interdependent, whereby disruption in these systems not only predisposes individuals to insomnia, hypersomnia, and sleep-disordered breathing but also fuels a vicious cycle of impaired brain clearance and neurodegeneration. Clinically, these results position sleep assessment as a valuable, accessible marker of glymphatic health and underscore the need to integrate sleep management into strategies aimed at preserving brain clearance and preventing neurological decline. Future research should prioritise longitudinal and standardised designs to clarify causality and evaluate interventions that simultaneously target sleep regulation and glymphatic function. Ultimately, our synthesis underscores a crucial message: nearly one in two individuals with glymphatic-related dysfunction experience sleep disorders. Protecting sleep may therefore represent one of the most effective strategies to preserve glymphatic integrity, mitigate neurovascular and neurodegenerative disease, and promote lifelong brain health.

## Figures and Tables

**Table 1:** PICOS framework for the inclusion and exclusion criteria of systematic review and meta-analysis.

Element	Inclusion Criteria	Exclusion Criteria
P—population	Human participants (any age) with sleep disorders and objective or proxy measures of glymphatic-related function (ePVS, WMHs, DTI-ALPS, ultrafast fMRI, or CSF/PET clearance markers)	Animal/in vitro studies; studies without glymphatic-related measures
I—Intervention	Presence of structural, functional, or biochemical evidence of impaired glymphatic function	None
C—Comparison	If available: individuals without glymphatic impairment or between subgroups (normal vs. abnormal glymphatic indices)	No comparator data
O—Outcomes	Occurrence or prevalence or proportion of participants diagnosed with any sleep disorder (insomnia, OSA, RBD, hypersomnia, etc.) based on validated diagnostic criteria	Studies without prevalence or standardized sleep disorder measures
S—Study Design	Observational (cross-sectional, cohort, or case–control)	Case reports, case series, protocol, reviews, conference abstracts, animal, and pre-clinical

ePVS, enlarged perivascular spaces; CSF, cerebrospinal fluid; DTI-ALPS, diffusion image analysis along perivascular spaces; fMRI, functional magnetic resonance imaging; OSA, obstructive sleep apnea’ RBD, Rapid eye movement sleep behaviour disorder; PET, positron emission tomography; WMHs, white matter hyperintensities.

**Table 2:** The global prevalence of sleep disorders based on study type, regions, and sleep disorder categories.

Subgroup		N	Sample Size	I^2^ (%)	Egger Test	Logit ES (95% CI)	Prevalence (%) (95% CI)
Study type	Cross-sectional	9	972	96.9	−1.70	−0.35 (−0.86–0.15)	41.34 (29.73–53.74)
	Case–control	10	1826	92.3	−1.95	−0.03 (−0.62–0.22)	49.25 (32.30–66.37)
Regions	Asia	11	2038	95	−1.54	−0.29 (−0.80–0.22)	42.8 (31.0–55.5)
	Europe	4	471	95.8	−2.07	−0.51 (−1.54–0.52)	37.5 (17.7–62.7)
	North America	3	196	93.3	−1.96	0.70 (−0.60–2.09)	66.8 (33.4–89.1)
	South America	1	93	*	*	−0.45 (−0.86–−0.04)	38.9 (29.8–49.0)
Sleep disorder categories	Insomnia Disorders	6	613	93.6	−0.95	−0.15 (−0.83–0.53)	46.3 (30.3–62.9)
	Hypersomnia/ EDS disorder	3	1249	98.1	−2.89	−0.85 (−2.31–0.62)	29.9 (9.0–65.0)
	Sleep-disordered breathing	3	241	94.9	−1.955	0.49 (−0.94–1.92)	37.9 (28.0–87.2)
	Parasomnias (REM-related)	5	515	93.3	−1.75	−0.36 (−1.13–0.41)	41.0 (24.3–60.1)
	Sleep-related movement disorders	1	120	*	*	0.30 (−0.06–0.66)	57.4 (48.5–65.9)
	Mixed/Comorbid	1	84	*	*	−0.11 (−0.53–0.31)	47.2 (37.0–57.3)

CI, confidence interval; ES, effect size; EDS, excessive daytime sleepiness; REM, rapid eye movement. I^2^ represents the percentage of variability due to between-study heterogeneity rather than chance; *p*-values within subgroups represent the test for heterogeneity (Cochran’s Q test); Random-effects models were used to calculate pooled prevalence estimates. * Statistics cannot be computed because this subgroup contains insufficient records.

**Table 3:** Meta-regression analyses of study-level moderators on the prevalence of sleep disorders.

Moderator	Estimate (β)	95% CI	*p*-Value	R^2^ (%)	I^2^ (%)
Publication year	−0.030	−0.389 to 0.329	0.86	0.00	95.6
Sample size	−0.002	−0.005 to −0.00007	0.44	14.2	93.1
Mean age	−0.030	−0.065 to 0.014	0.19	3.1	94.1

CI, confidence interval.

## Data Availability

Data is available with the corresponding author upon request.

## Supplementary Materials

The following supporting information can be downloaded at: https://www.mdpi.com/article/10.3390/biology15040309/s1, Table S1: Detailed Search Strategy used for Literature Searching. Table S2: Quality check using Newcastle-Ottawa Scale (NOS) adapted for case-control, cohort, and cross-sectional studies. Table S3: Characteristics of the extracted data from the eligible papers included.

## Abbreviations

The following abbreviations are used in this manuscript:

DTI-ALPS	Diffusion tensor imaging analysis along perivascular space
fMRI	Functional magnetic resonance imaging
CSF	Cerebrospinal fluid
PET	Positron emission tomography
AD	Alzheimer’s disease
Aβ	Amyloid beta
OSA	Obstructive sleep apnea
PD	Parkinson’s disease
ePVS	Enlarged perivascular spaces
SWS	Slow wave sleep
ISF	Interstitial fluid
MRI	Magnetic resonance imaging
PICOS	Population, Intervention (or Exposure), Comparison, Outcomes, and Study design
WMHs	White matter hyperintensities
NOS	Newcastle–Ottawa Scale
REML	Restricted maximum likelihood
CSVD	Cerebral small vessel disease
NT1	Narcolepsy type 1
SSADHD	Succinic semialdehyde dehydrogenase deficiency
PLMS	Periodic limb movements in sleep
EDS	Excessive daytime sleepiness
PSQI	Pittsburgh Sleep Quality Index
ICSD-3	International Classification of Sleep Disorders, Third Edition
AASM	American Academy of Sleep Medicine
PSG	Polysomnography
ESS	Epworth Sleepiness Scale
CSHQ	Children’s Sleep Habits Questionnaire
REM	Rapid eye movement
AQP-4	Aquaporin-4
BBB	Blood–brain barrier
