# Supplementary material for: The Prevalence of Sleep Disorders in Populations with Glymphatic Dysfunction: A Systematic Review and Meta-Analysis
Source: Biology (Basel). 2026 Feb 10;15(4):309. doi: 10.3390/biology15040309 (PMC12937776; doi:10.3390/biology15040309)
Supplement: Supplementary file 1 [file biology-15-00309-s001.zip › biology-4136561-supplementary.pdf]

Supplementary Table S1: Detailed Search Strategy used for Literature Searching

Database	Search Strings Used	Publication Years	Number of Studies Identified
PubMed	((((((((((((((sleep disorder[Title/Abstract]) OR (insomnia[Title/Abstract])) OR (Sleep-related breathing disorders[Title/Abstract])) OR (Central disorders of hypersomnolence[Title/Abstract])) OR (Circadian rhythm sleep-wake disorders[Title/Abstract])) OR (parasomnias[Title/Abstract])) OR (Sleep-related movement disorders[Title/Abstract])) OR (sleep disorders[MeSH Terms])) OR (insomnia[MeSH Terms])) OR (Sleep-related breathing disorders[MeSH Terms])) OR (Central disorders of hypersomnolence[MeSH Terms])) OR (Circadian rhythm sleep-wake disorders[MeSH Terms])) OR (parasomnias[MeSH Terms])) OR (Sleep-related movement disorders[MeSH Terms]) AND (humans[Filter])) AND ((((((((prevalence[Title/Abstract]) OR (epidemiology[Title/Abstract])) OR (frequency[Title/Abstract])) OR (burden[Title/Abstract])) OR (prevalence[MeSH Terms])) OR (epidemiology[MeSH Terms])) OR (frequency[MeSH Terms])) OR (burden[MeSH Terms]) AND (humans[Filter]))) AND ((((((((((((((glymphatic system[Title/Abstract]) OR (glymphatic[Title/Abstract])) OR (perivascular space[Title/Abstract])) OR (Virchow-Robin space[Title/Abstract])) OR (PVS[Title/Abstract])) OR (ePVS[Title/Abstract])) OR (meningeal lymphatic[Title/Abstract])) OR (glymphatic system[MeSH Terms])) OR (glymphatic[MeSH Terms])) OR (perivascular space[MeSH Terms])) OR (Virchow-Robin space[MeSH Terms])) OR (PVS[MeSH Terms])) OR (ePVS[MeSH Terms])) OR (meningeal lymphatic[MeSH Terms]) AND (humans[Filter]))	N/A	12
WoS (ESCI) & (SCI-EXPANDED)	TS=(("sleep disorder*" OR “insomnia*” OR "Sleep-related breathing disorders*" OR "Central disorders of hypersomnolence*" OR "Circadian rhythm sleep-wake disorders*" OR “parasomnias*” OR "Sleep-related movement disorders*") AND (“prevalence*” OR “epidemiology*” OR “frequency*” OR “burden*”) AND ("glymphatic system*" OR “glymphatic*” OR "perivascular space*" OR "Virchow-Robin space*" OR “PVS*” OR “ePVS*” OR "meningeal lymphatic*"))	N/A	56
SCOPUS	((TITLE-ABS-KEY ("sleep disorder") OR TITLE-ABS-KEY (insomnia) OR TITLE-ABS-KEY ("Sleep-related breathing disorders") OR TITLE-ABS-KEY ("Central disorders of hypersomnolence") OR TITLE-ABS-KEY ("Circadian rhythm sleep-wake disorders") OR TITLE-ABS-KEY (parasomnias) OR TITLE-ABS-KEY ("Sleep-related movement disorders"))) AND ((TITLE-ABS-KEY (prevalence) OR TITLE-ABS-KEY (epidemiology) OR TITLE-ABS-KEY (frequency) OR TITLE-ABS-KEY (burden))) AND ((TITLE-ABS-KEY ("glymphatic system") OR TITLE-ABS-KEY (glymphatic) OR TITLE-ABS-KEY ("perivascular space") OR TITLE-ABS-KEY ("Virchow-Robin space") OR TITLE-ABS-KEY (PVS) OR TITLE-ABS-KEY (ePVS) OR TITLE-ABS-KEY ("meningeal lymphatic")))	N/A	63
ProQuest	("sleep disorder" OR “insomnia” OR "Sleep-related breathing disorders" OR "Central disorders of hypersomnolence" OR "Circadian rhythm sleep-wake disorders" OR “parasomnias” OR "Sleep-related movement disorders") AND (“prevalence” OR “epidemiology” OR “frequency” OR “burden”) AND ("glymphatic system" OR “glymphatic” OR "perivascular space" OR "Virchow-Robin space" OR “PVS” OR “ePVS” OR "meningeal lymphatic")<br><br>Filter: (Scholarly Journals OR Reports OR Working Papers) NOT (Books AND Dissertations & Theses AND Wire Feeds AND Conference Papers & Proceedings AND Magazines AND Trade Journals)		555

N/A= not applicable, ESCI = Emerging Sources Citation Index, SCI-EXPANDED = Science Citation Index Expanded, WoS = Web of Science.

Supplementary Table S2: Quality check using Newcastle-Ottawa Scale (NOS) adapted for case-control, cohort, and cross-sectional studies.

Study Cohort	Selection				Comparability		Outcome			Total
	Representative of the exposed cohort	Selection of external control	Ascertainment of exposure	Outcome of interest does not present at the start of the study	Main factor	Additional factor	Assessment of outcomes	Sufficient follow-up time	Adequacy of follow-up	
(Del Brutto et al., 2022)	1	0	1	1	1	0	1	1	1	8/9
(Sotgiu et al., 2023)	1	1	1	0	1	1	1	1	1	8/9
(Jarvela et al., 2022)	1	1	1	1	1	0	1	1	1	8/9
(Tokatly et al., 2024)	1	1	1	0	1	1	1	1	1	8/9
Park et al., 2023	1	1	1	1	1	0	1	1	0	7/9
Roura et al., 2025	1	1	1	1	1	0	1	1	0	7/9
Marecek et al., 2025	1	1	1	1	1	0	1	1	0	7/9
Bae et al., 2023	1	1	1	1	1	0	1	1	1	8/9
Si et al., 2020	1	1	1	1	1	1	1	1	0	8/9
Lee et al., 2021	1	1	1	1	1	0	1	1	0	7/9
**Study Cross-sectional**	**Selection**			**Control of confounders**		**Outcome**				**Total**
	**Representative of the sample**	**Sample size**	**Non-respondents**	**Study controls for the most important factor**	**Study controls for any additional factor**	**Assessment of outcomes**		**Statistical test**	**Ascertainment of the outcome measurement (validated measurement tool)**	
(Du et al., 2024)	1	1	0	0	0	1	1	1	1	6/9
(Gui et al., 2024)	1	1	0	0	0	1	1	1	1	6/9
(Jia et al., 2021)	1	1	0	1	0	1	1	1	1	7/9
(Wang et al., 2020)	1	1	0	1	0	1	1	1	1	7/9
(Kang et al., 2018)	1	1	0	0	1	1	1	1	1	7/9
(Lao et al., 2022)	1	1	0	1	0	1	1	1	1	7/9
(Opel et al., 2019)	1	0	0	1	0	1	1	1	1	6/9
(Zhao et al., 2022)	1	1	0	1	1	1	1	1	1	8/9
(Zhao et al., 2023)	1	1	0	1	1	1	1	1	1	8/9

Notes: Newcastle-Ottawa Scale contains 8 items within 3 domains, and the total maximum score is 9.
A study with a score from 7-9 = high quality, 4-6 =moderate quality, and 0-3 =very high risk of bias.

Supplementary Table S3: Characteristics of the extracted data from the eligible papers included.

First author/s. year	Country	Type of study	Population characteristics	Type of sleep disorder and diagnostic method	Sample size	Occurrence (%) [a]	Glymphatic method and measurement
Kang et al., 2018 [27]	South Korea	Cross-sectional case–control	Adults evaluated for sleep disturbance<br><br>Mean age PLMS: 61.7 ± 14.4 years; Mean age control: 58.2 ± 14.8 years	Disorder: PLMS<br><br>Method: Overnight full PSG scored per WASM/IRLSSG standards	60	51.67	ePVS, specifically in the basal ganglia
Opel et al., 2019 [24]	USA	Cross-sectional	Individuals who had both overnight PSG and brain MRI available; subsets had medically confirmed TBI.<br><br>Mean age: 57.1 ± 14.2 years (26-88 years old)	Disorder: Sleep apnea,<br><br>Method: Overnight PSG, ISI, and ESS	38	89.5	ePVS
Wang et al., 2020 [29]	China	Cross-sectional	Patients with CSVD (50-70 years old).<br><br>Mean age CSVD: 60.1 ± 8.4 years; Controls: 61.8 ± 10.2 years.	Disorder: Non-breathing-related sleep fragmentation; includes insomnia symptoms/disorder.<br><br>Method: Overnight PSG, AASM criteria measures: ArI, WASO, SE, sleep stages (N1–3, REM), PLMSI, PLMAI; sleep quality via PSQI	108	Insomnia: 64.3% of CSVD patients; OSAS in screened: 9.1%	Basal ganglia ePVS severity (semi-quantitative rating scale) and WMHs severity;

Jia et al., 2021 [30]	China	Cross-sectional	Adults aged 18–60 undergoing routine health screening.<br><br>Mean age: 31.72 ± 11.86 years	Disorder: OSA categorized by severity.<br><br>Method: PSG scored per AASM v2.4 criteria<br><br>Control (AHI,5/h); Mild (AHI 5-15/h); Moderate (AHI 15-30/h); Severe (AHI >30/h)	107	58.87	ePVS counts
Del Brutto et al., 2022 [31]	Ecuador	Case-control	Community-dwelling adults with long COVID and poor sleep quality<br><br>Mean age: 69.4 ± 6.5 years	Persistent poor sleep quality post–COVID measured by PSQI	93	38.81	Progression of ePVS
Järvelä et al., 2022 [32]	Finland	Case-control	Patients with NT1 and age-/sex-matched healthy controls<br><br>NT1: 28.1 ± 8.9 years; controls: 28.2 ± 8.9 years	Disorder: Narcolepsy type 1<br><br>Method: confirmed via ICSD-3 criteria (including cataplexy confirmation)	66	33.33	Brain pulsation dynamics proxy for CSF/glymphatic flow.<br><br>Ultrafast fMRI (MREG) acquisition (~10 Hz temporal resolution)
Lao et al., 2022 [33]	USA	Cross-sectional	Adults with DS aged ~50 years, categorized as cognitively stable (64%), MCI-DS (23%), possible AD (5%), or definite AD (8%).<br><br>Mean age: 50 ± 8 years	Disorder: OSA<br><br>Method: reported by caregiver or medical record (no objective PSG data).	116	39	ePVS quantified visually and WMHs volume
Zhao et al., 2022 [26]	China	Cross-sectional,	Community-dwelling older adults aged 55–85 years<br><br>Mean age: 65.58 ± 6.46 years	Disorder: EDS<br><br>Method: ESS (EDS is defined as an ESS score > 10)	1076	Overall: 10%; among CSVD subjects: 20%	CSVD neuroimaging markers, including WMHs, lacunes, CMBs, and ePVS (basal ganglia)
Sotgui et al., 2023 [34]	Italy	Case–control	Children (2-7 years old) with ASD vs control	Disorder: Insomnia	136	17.64	White-matter perivascular spaces quantification.

			patients with other neuropsychiatric disorders.<br><br>The median age in the total cohort was 4 years (IQR: 2–6), 4 years in the NP/Non-ASD, and 3 years in the ASD group.	Method: Diagnosed with a parent-report sleep screening instrument designed, the CSHQ, and wake and sleep EEG features			
Tokatly et al., 2023 [27]	USA	Case–control	SSADHD patients with genetically confirmed succinic semialdehyde dehydrogenase deficiency, healthy non-related control individuals<br><br>Mean age SSADHD: 13.4 ± 9.7 years<br><br>Mean age control: 18.2 ± 9.8 years	Disorder: Sleep disturbances<br><br>Method: CSHQ with a total score ≥41 indicates clinically meaningful sleep disturbances	42	Overall SSADHD group: 67% had CSHQ ≥ 41; severe ePVS group: 80%; mild/moderate ePVS: 36%	(i) ePVS burden via visual MRI scoring; (ii) GABA levels via MRS and plasma measurements
Zhao et al., 2023 [25]	China	Cross-sectional	Patients with CSVD and healthy controls<br><br>Controls: 61.5 ± 6.1 years; CSVD-GS: 64.9 ± 6.4 years; CSVD-PS: 64.3 ± 7.1 years	Disorder: Sleep disturbance<br><br>Method: PSQI	99	36.36	ePVS quantified in the basal ganglia
Du et al., 2024 [22]	China	Cross-sectional	Adults post-Omicron infection<br><br>Mean age HC: 41.85 ± 13.90<br>Mean CIA: 40.04 ± 11.75	Disorder: Insomnia (chronic and new-onset, subjective)<br><br>Method: ICSD-3 criteria for insomnia (subjective self-report)	135	59.26	DTI-ALPS inde
Gui et al., 2024 [23]	China	Cross-sectional	Patients with PD (non-motor symptoms varied)<br><br>Mean age PD: 71.71 ± 7.49 years; Mean age Controls: 70.34 ± 7.57 years	Disorder: Insomnia and RBD<br><br>Method: Standardized scales, plus EDS	87	Insomnia: 57.14%; EDS: 26.53%; RBD: 46.94%	ePVS count in the and DTI-ALPS

Park et al., 2023 [35]	South Korea	Case-control	Patients with primary RLS and healthy controls<br><br>Mean age: 56.5 ± 7.4	Disorder: Restless leg syndrome<br><br>Method: International RLS scale, PSQI, and ISI	120	57.5	DTI-ALPS index
Roura et al., 2025 [36]	Spain	Case-control	iRBD patients and HC<br><br>Mean age: 68.5 ± 6.5	Disorder: iRBD<br><br>Method: PSG	85	73	ePVS and DTII-ALPS
Marecek et al., 2025 [37]	Czech Republic	Case-control	iRBD and PD<br><br>Mean age: 60 ± 10	Disorder: iRBD<br><br>Method: ICSD-3 and PSG	184	31	ePVS and DTII-ALPS
Bae et al., 2023 [38]	South Korea	Case-control	iRBD and PD<br><br>Mean age: 72.5	Disorder: iRBD<br><br>Method: PSG	60	33.33	SPECT and DTI-ALPS
Si et al., 2020 [39]	China	Case-control	iRBD and PD<br><br>Mean age: 60 ± 9	Disorder: iRBD<br><br>Method: PSG	150	22	ePVS counts
Lee et al., 2021 [40]	South Korea	Case-control	iRBD patients and HC<br><br>Mean age: 68.3 ± 6.7	Disorder: iRBD<br><br>Method: PSG	36	50	DTI-ALPS

[a] Point prevalence of sleep disorder associated with glymphatic dysfunction (and its proxy) (%). AASM, American Academy of Sleep Medicine; AD, Alzheimer's disease; AHI, Apnea-hypopnea index; ArI, Arousal index; CSHQ, Children's Sleep Habits Questionnaire; CSVD, Cerebral small vessel disease; DS, Down syndrome; DTI-ALPS, Diffusion tensor image analysis along the perivascular space; EDS, Excessive daytime sleepiness; EEG, Electroencephalography; ePVS, enlarged perivascular spaces; ESS, Epworth sleepiness scale; HC, healthy control; ICSD-3, International classification of sleep disorders – third edition; iRBD, isolated rapid eye movement sleep behaviour disorder; IRLSSG, International Restless Legs Syndrome Study Group; ISI, Insomnia severity index; MCI-DS, Mild cognitive impairment down syndrome; MRI, magnetic resonance imaging; MRS, magnetic resonance spectroscopy; NT1, narcolepsy type 1; PD, Parkinson's disease; PLMAI; periodic limb movement arousal index; PLMS, periodic limb movements; PLMSI, Periodic limb movements during sleep index; PSG, polysomnography; PSQI, Pittsburgh sleep quality index; REM, rapid eye movement; SE, Sleep efficiency; SPECT, single-photon emission computed tomography; SSADHD, succinic semialdehyde dehydrogenase deficiency; TBI, traumatic brain injury; WASM, World association of sleep medicine; WASO, wake after sleep onset; WMHs, white matter hyperintensities.
